# Study on the effect of EMD386088, a 5-HT_6_ receptor partial agonist, in enhancing the anti-immobility action of some antidepressants in rats

**DOI:** 10.1007/s00210-017-1431-y

**Published:** 2017-10-27

**Authors:** Magdalena Jastrzębska-Więsek, Agata Siwek, Anna Partyka, Marcin Kołaczkowski, Maria Walczak, Magdalena Smolik, Gniewomir Latacz, Katarzyna Kieć-Kononowicz, Anna Wesołowska

**Affiliations:** 1 Department of Clinical Pharmacy, Jagiellonian University Medical College, 9 Medyczna Street, 30-688 Cracow, Poland; 2 Department of Pharmacobiology, Jagiellonian University Medical College, 9 Medyczna Street, 30-688 Cracow, Poland; 3 Department of Pharmaceutical Chemistry, Jagiellonian University Medical College, 9 Medyczna Street, 30-688 Cracow, Poland; 4 Adamed Ltd., Pienków 149, 05-152 Czosnów, Poland; 5 Department of Toxicology, Jagiellonian University Medical College, 9 Medyczna Street, 30-688 Cracow, Poland; 6 Department of Technology and Biotechnology of Drugs, Jagiellonian University Medical College, 9 Medyczna Street, 30-688 Cracow, Poland

**Keywords:** EMD386088, 5-HT_6_ receptor partial agonist, Antidepressants, Pharmacokinetic, Metabolic stability

## Abstract

The effect of some antidepressants co-administered with EMD386088 in the modified forced swim test in rats was investigated. Additionally, the pharmacokinetics, metabolic stability, and the effect of EMD386088 on P450 cytochromes were determined. Intraperitoneal (i.p.) coadministration of EMD386088 (2.5 mg/kg) and imipramine (15 mg/kg), reboxetine (5 mg/kg), moclobemide (10 mg/kg), or bupropion (10 mg/kg) evoked significant antidepressant-like activity, whereas no effect was observed after joint administration of EMD386088 with s-citalopram (10 mg/kg). Pharmacokinetic in vivo investigation showed a rapid absorption of EMD386088 (2.5 and 5 mg/kg) with *t*_1/2_ = 67 min (*t*_max_ = 5 min). Large volume of distribution (*V*_d_/F = 102 L/kg) indicated its penetration into peripheral compartments. The most active coadministration of EMD386088 (2.5 mg/kg) with imipramine (15 mg/kg) resulted in slower absorption of the compound (*C*_max_ = 60 min) and decrease in the volume of distribution (*V*_d_/F = 32.2 L/kg). EMD386088 penetrates the blood–brain barrier with a high brain/plasma ratio of about 19 (2.5 mg/kg) and 7.5 for coadministration with imipramine. The in silico and in vitro studies on EMD386088 metabolic stability showed the dehydrogenation of tetrahydropyridine moiety as its main metabolic pathway. EMD386088 did not influence on CYP3A4 activity, and it has been classified as a very weak CYP2D6 inhibitor (*IC*_*50*_ = 2.25 μM). The results obtained from the forced swim test in rats indicate that an activation of 5HT_6_ receptor may facilitate antidepressant-like activity of some antidepressants. The pharmacokinetic results suggest that the interaction between EMD386088 and imipramine could not have been pharmacokinetic in nature.

## Introduction

The serotonin-6 (5-HT_6_) receptors belong to serotonin receptors that are positively coupled to the G_s_ protein and stimulate adenylate cyclase activity (Monsma et al. 1993; Ruat et al. 1993; Plassat et al. 1993). Their distribution in the limbic areas and ability for different neurotransmission system modulations (i.e., cholinergic, glutamatergic, and γ-aminobutyric acid-ergic (GABAergic)) indicate that 5-HT_6_ receptors are involved in mood and cognitive processes (Ward et al. 1995; Gérard et al. 1996; Yoshioka et al. 1998; Takamori et al. 2001). Literature preclinical data show that 5-HT_6_ receptors have an important function in regulating mood since both agonists/partial agonists (i.e., WAY181187, WAY208466, EMDT, ST1936) and antagonists (i.e., SB271046, SB258585, SB399885) of these receptors possess antidepressant-like activity (Svenningsson et al. 2007; Wesołowska et al. 2007; Wesołowska 2008; Hirano et al. 2009; Carr et al. 2011; Nikiforuk et al. 2011; Jastrzębska-Więsek et al. 2014, 2015). At present, it is difficult to indicate molecular mechanisms through which both activation and blockade of these receptors are able to evoke the same antidepressant effect observed in animals.

The compound EMD386088 has been presented by Mattsson et al. (2005) as a 5-HT_6_ receptor agonist (EC_50_ = 1.0 nM). Recently obtained in vitro results demonstrated that EMD386088 behaves as a potent 5-HT_6_ receptor partial agonist (Jastrzębska-Więsek et al. 2013). It also has no affinity for α_1_-, α_2_-, and β_1_-adrenoceptors, dopamine D_2_, dopamine D_3_, GABA_A_, opioid μ receptors, and serotonin (5-HT) transporter (Jastrzębska-Więsek et al. 2013). Previous findings have indicated that EMD386088 exerts an antidepressant-like effect after acute, sub-chronic (three times during 24 h in the forced swim test (FST)) and chronic (once daily for 14 days) systemic administration in the olfactory bulbectomy model in rats (Jastrzębska-Więsek et al. 2015). Previously obtained data confirm the importance of dopaminergic system activation in antidepressant-like activity of EMD386088 after acute administration, since this effect, observed in the FST, was abolished by the preferential D_1_- and D_2_-like receptor subfamily antagonists SCH23390 and sulpiride, respectively (Jastrzębska-Więsek et al. 2016). In the ex vivo neurochemical studies, EMD386088 changed only the dopamine metabolism and activity of dopaminergic system in all investigated brain structures, i.e., hippocampus, nucleus accumbens, and striatum but there was not observed effect on the noradrenergic- or serotonergic system. In functional in vitro study, EMD386088 showed significant affinity for dopamine transporter (DAT). These all data confirm that antidepressant-like activity of EMD386088 in FST may be connected with the activation of dopaminergic system, while neither noradrenergic nor serotonergic ones are involved in its effect (Jastrzębska-Więsek et al. 2016).

We designed a modified version of FST in rats to evaluate the interaction between EMD386088 and some antidepressants with a different mechanism of action. Antidepressant drugs, chosen for the study, included the 5-HT/noradrenaline reuptake inhibitor imipramine, the selective noradrenaline reuptake inhibitor reboxetine, the selective 5-HT reuptake inhibitor s-citalopram, the dopamine reuptake inhibitor bupropion, and the monoamine oxidase-A inhibitor moclobemide. All compounds were given as non-active doses, chosen from the results of our earlier studies (Wesołowska and Nikiforuk 2007; Jastrzębska-Więsek et al. 2015).

Since pharmacokinetic studies are an important part of tests performed for new compounds with proven biological activity, we decided to investigate the pharmacokinetic profile of EMD386088 through a single i.p. administration and by coadministration with imipramine. We also chose to determine in vitro, the effect of EMD386088 on recombinant isoenzymes CYP3A4 and CYP2D6 of P450 cytochrome, since their potential inhibition could be involved in antidepressant effects observed in FST. Metabolic stability of EMD386088 was also evaluated in silico by MetaSite software and in vitro using rat liver microsomes (RLMs).

## Material and methods

### Animals

The experiments were performed on male Wistar rats (250–300 g) purchased from Charles River Laboratories (Germany). The animals were housed for a period of 6 days in polycarbonate Makrolon type 3 cages (dimensions 26.5 × 15 × 42 cm) in an environmentally controlled room (ambient temperature 21 ± 2 °C; relative humidity 50–60%; 12:12 light:dark cycle, lights on at 8:00), in groups of four rats. Standard laboratory food (LSM-B) and filtered water were freely available.

In behavioral experiments, animals were assigned randomly to treatment groups. All the tests were performed by two observers unaware of the treatment applied between 9:00 and 14:00 on separate groups of animals.

A pharmacokinetic study was carried out for EMD386088 and imipramine following their single and combined i.p. administrations to rats. Blood samples were collected at 0 min (predose), 5 min, 15 min, 30 min, 60 min, 120 min, and 240 min after injections. The blood was collected from right ventricle under anesthesia using a mixture of ketamine (50 mg/kg)/xylasine (8 mg/kg) in the ratio of 3:1, and 0.025 mL of a mixture for 10 g body weight was injected i.p. 10 min before planned blood collection. Blood and brain samples were collected under general anesthesia induced by i.p. injections of ketamine (50 mg/kg)/xylasine (8 mg/kg). The blood samples were taken into heparinized tubes, immediately centrifuged at 3500 rpm for 10 min, and plasma was collected. After the blood was drawn, the rat’s spinal cord was cut and the cerebral tissue was removed, and after dissection washed in PBS saline. The brain and plasma samples were immediately frozen at − 80 °C for LC/MS/MS analysis.

Procedures involving animals and their care were conducted in accordance with current European Community and Polish legislation on animal experimentation. Additionally, all efforts were made to minimize animal suffering and to use only the number of animals necessary to produce reliable scientific data. The experimental protocols and procedures described in this manuscript were approved by the IV Local Ethics Commission in Warsaw (no 40/2008) and were in accordance with EU Directive 2010/63/EU and the 1996 NIH Guide for the Care and Use of Laboratory Animals.

### Drugs

The following drugs were used: 5-chloro-2-methyl-3-(1,2,3,6-tetrahydro-4-pyridinyl)-1*H*-indole hydrochloride (EMD386088) and s-citalopram were synthesized by Adamed (Pieńków, Poland), imipramine (Sigma Aldrich, Germany), moclobemide (Sigma Aldrich, Germany), reboxetine (Ascent, UK), bupropion (Sigma Aldrich, Germany), xylazine (Sigma-Aldrich, USA), ketamine (Vetoquinol, Poland). All drugs were dissolved in distilled water immediately before administration in a volume of 2 mL/kg. All the compounds were administered intraperitoneally (i.p.). EMD386088, imipramine, and s-citalopram were given 30 min before the test, while the remaining compounds were injected 60 min before. Control animals received vehicle according to the same schedule.

### Behavioral experiments

#### Forced swim test in rats

The experiment was carried out according to the modified by Detke et al. (1995) method of Porsolt et al. (1978). On the first day of an experiment, the animals were gently individually placed in Plexiglas cylinders (40 cm high, 18 cm in diameter) containing 30 cm of water maintained at 23–25 °C for 15 min. On removal from water, the rats were placed for 30 min in a Plexiglas box under 60-W bulb to dry. On the following day (24 h later), the rats were re-placed in the cylinder and the total duration of immobility, swimming, and climbing was recorded during the whole 5-min test period. The swimming behavior entailed active swimming motions, e.g., moving horizontally around in the cylinder. Climbing activity consisted of upward directed movements of the forepaws along the side of the swim chamber, and immobility was assigned when no additional activity was observed other than that necessary to keep the rat’s head above the water (Detke et al. 1995). Fresh water was used for each animal.

#### Open field test in rats

The experiment was performed using Motor Monitor System (Campden Instruments, Ltd., UK) consisted of two SmartFrame Open Field stations (40 × 40 × 38 cm) with 16 × 16 beams, located in sound attenuating chambers and connected to PC software by control chassis. Individual vehicle- or drug-injected animals were gently placed in the center of the station. An automated motor monitor system recorded ambulation (the number of crossings in X and Y axis), the number of rearing episodes, and total distance covered by a rat for 5 min.

#### Statistical analysis

The data of behavioral studies were evaluated using two-way analysis of variance (ANOVA) followed by Bonferroni’s post hoc test, *p* < 0.05 was considered significant.

### Pharmacokinetic study in rats

Pharmacokinetic parameters were calculated by a non-compartmental approach from the average concentration values, using Phoenix WinNonlin software (Certara, Princeton, NJ 08540 USA). First order elimination rate constant (λ_z_) was calculated by linear regression of time versus log concentration. Next, the area under the mean plasma and brain concentration versus time curve (AUC_0 → t_) was estimated using the log-linear trapezoidal rule (Eq. 1), where *C*_n_ is the concentration of last sampling of the compound.

1 $$ {\mathrm{AUC}}_{0\to \mathrm{t}}={\sum}_{i=1}^n\left(\left({C}_i+{C}_{i+1}\right)/2\right)\bullet \left({t}_{i+1}-{t}_i\right)+{C}_{\mathrm{n}}/{\uplambda}_{\mathrm{z}} $$

Area under the first-moment curve (AUMC_0→t_) was estimated by calculation of the total area under the first-moment curve using the Eq. 2, where *t*_n_ is the time of last sampling.

2 $$ {\mathrm{AUMC}}_{0\to \mathrm{t}}=\sum \limits_{\mathrm{i}=1}^n\left(\left({t}_i\bullet {C}_i+{t}_{i+1}\bullet {C}_{i+1}\right)/2\right)\bullet \left({t}_{i+1}-{t}_i\right)+\left({t}_{\mathrm{n}}\bullet {C}_{\mathrm{n}}\right)/{\uplambda}_{\mathrm{z}}+{C}_{\mathrm{n}}/{\uplambda}_{\mathrm{z}}^2 $$

Mean residence time (MRT) was calculated as follows:

3 $$ \mathrm{MRT}=\frac{{\mathrm{AUMC}}_{0\to t}}{{\mathrm{AUC}}_{0\to t}} $$

Total clearance (Cl_T_) was calculated as follows:

4 $$ {\mathrm{Cl}}_{\mathrm{T}}=\frac{F\cdot {D}_{\mathrm{i}.\mathrm{p}.}}{{\mathrm{AUC}}_{0\to t}} $$

Volume of distribution (*V*_d_) was calculated as:

5 $$ {V}_{\mathrm{d}}=\frac{F\cdot {D}_{\mathrm{i}.\mathrm{p}.}}{\lambda_{\mathrm{z}}\cdot {\mathrm{AUC}}_{0\to t}} $$

where *D*_i.p._ is an i.p. dose of EMD386088.

#### Analytical method

The quantification of studied compounds in plasma and brain samples was done using HPLC Agilent 1100 system (Agilent Technologies, Waldbronn, Germany) coupled to the triple quadrupole mass spectrometer API 2000 (ABSciex, Framingham, Massachusetts, USA) equipped with an electrospray ion source. After preparation, the samples were injected (20 μL) onto XBridge C_18_ (2.1 mm × 30 mm, 3.5 μm, Waters, Milford, Massachusetts, USA) analytical column. The mobile phase consisted of ACN with 0.1% formic acid (A) and water with 0.1% formic acid (B) were delivered in gradient elution started with 90% of eluent B, increasing during 2 min to 90% of eluent A, maintained to 3 min, and then returned during 5 min to 90% of eluent B, and maintained 90% of eluent B during 5 min at a flow rate of 300 μL/min. The total time of analysis was 15 min. Electrospray ionization process was performed in positive ionization and the data acquisition was carried out in multiple reaction monitoring mode (MRM) for EMD386088 and its internal standard (nebivolol). The ion spray source settings were as follows: spray voltage: 5 kV, heater temperature: 350 °C, curtain gas: 15 psi, source gas 1: 35 psi, source gas 2: 10 psi. The ions measured were m/z 247.3 (Q1) and m/z 168.2 (Q3) for EMD386088, and m/z 406.2 (Q1) and m/z 151.1 (Q3) for IS.

#### Preparation of standard solutions

An amount of 10 mg of EMD386088 was accurately weighted and quantitatively transferred into the 10-mL volumetric flask using MeOH. After the salts dissolving, flask was filled to the 10-mL mark with MeOH obtaining 1 mg/mL of an analyte. Further dilutions were performed using MeOH to prepare working standard solutions of the analyte at the following concentrations: 0.025, 0.05, 0.1, 0.25, 0.5, 1.0, 2.5, 5.0, 10, 25, and 50 μg/mL for calibration curve samples (CC) and 0.025, 0.075, 2.2, and 4.5 μg/mL for quality control samples (QC). To prepare samples for calibration curve or quality control samples, 45 μL of matrix (plasma or brain homogenate) were spiked with 5 μL of an internal standard solution obtaining the final concentration of 100 ng/mL and 5 μL of standards working solutions at needed CC or QC concentration levels. After standards solution addition, samples were mixed and purified.

#### Sample preparation

A sample volume of 50 μL of plasma or brain homogenate was transferred into the clean Eppendorf tube and spiked with 5 μL of IS solution (1 μg/mL) obtaining the final concentration of 100 ng/mL. After 5 min of mixing (1500 rpm), proteins were precipitated using 150 μL ACN. After 10 min of samples shaking (1500 rpm), the incubation step was performed (10 min, 4 °C). Next, samples were centrifuged (10,000 rpm, 10 min, 4 °C) and the supernatant was transferred into chromatographic vial for LC/MS/MS analysis. The brain homogenate was prepared maintaining the tissue: phosphate-buffered saline (PBS) at a ratio of 1:5. The homogenization was carried out employing IKA® T10 Basic ULTRA-TURRAX disperser (IKA Werke GmbH & Co. KG, Staufen, Germany). After homogenization, 50 μL of sample was collected for further preparation. All samples were stored on ice during the preparation process.

### Metabolic stability of EMD386088 investigations

#### In silico investigation

The in silico study was performed by MetaSite 5.1.1 provided by Molecular Discovery Ltd. The highest metabolism probability sites and the prediction of the structures of metabolites were analyzed during this study by liver computational model (Cruciani et al. 2005).

#### In vitro investigation

Commercial, pooled, rat liver microsomes (RLMs) were purchased from Sigma-Aldrich (St. Louis, USA). The biotransformations were carried out using 1 mg/mL of RLMs in 200 μL of reaction buffer containing 0.1 M Tris-HCl (pH 7.4) and EMD386088 with final volume 50 μM. The reaction mixture was preincubated at 37 °C for 5 min and then, the reaction was initiated by adding 50 μL of NADPH Regeneration System (Promega, Madison, WI, USA). The reaction was terminated after 30 or 120 min by the addition of 200 μL of cold methanol. The mixture was next centrifuged at 14000 rpm for 15 min and the UPLC/MS analysis of the supernatant was performed. Mass spectra was recorded on UPLC/MS system consisted of a Waters Acquity UPLC (Waters, Milford, USA), coupled to a Waters TQD mass spectrometer (electrospray ionization mode ESI-tandem quadrupole).

### Influence on recombinant human CYP3A4 and 2D6 P450 cytochromes

The luminescent CYP3A4 P450-Glo™ and CYP2D6 P450-Glo™ assays and protocols were provided by Promega (Madison, WI, USA) (Cali et al. 2006). The reference drugs ketoconazole (KE) and quinidine (QD) were obtained from Sigma-Aldrich (St. Louis, USA). The enzymatic reactions were performed in white polystyrene, flat-bottom Nunc™ MicroWell™ 96-Well Microplates (Thermo Scientific, Waltham, MA USA). The luminescence signal was measured with a microplate reader in luminescence mode (EnSpire, PerkinElmer, Waltham, MA USA). The IC_50_ values of KE and QD were determined and calculated as we described previously (Sadek et al. 2016). The final concentrations of EMD 386088 were similar for both CYP3A4 and CYP2D6 assays from 0.025 to 25 μM.

## Results

### Interaction between EMD386088 and antidepressant drugs in the FST

**Fig. 1:**
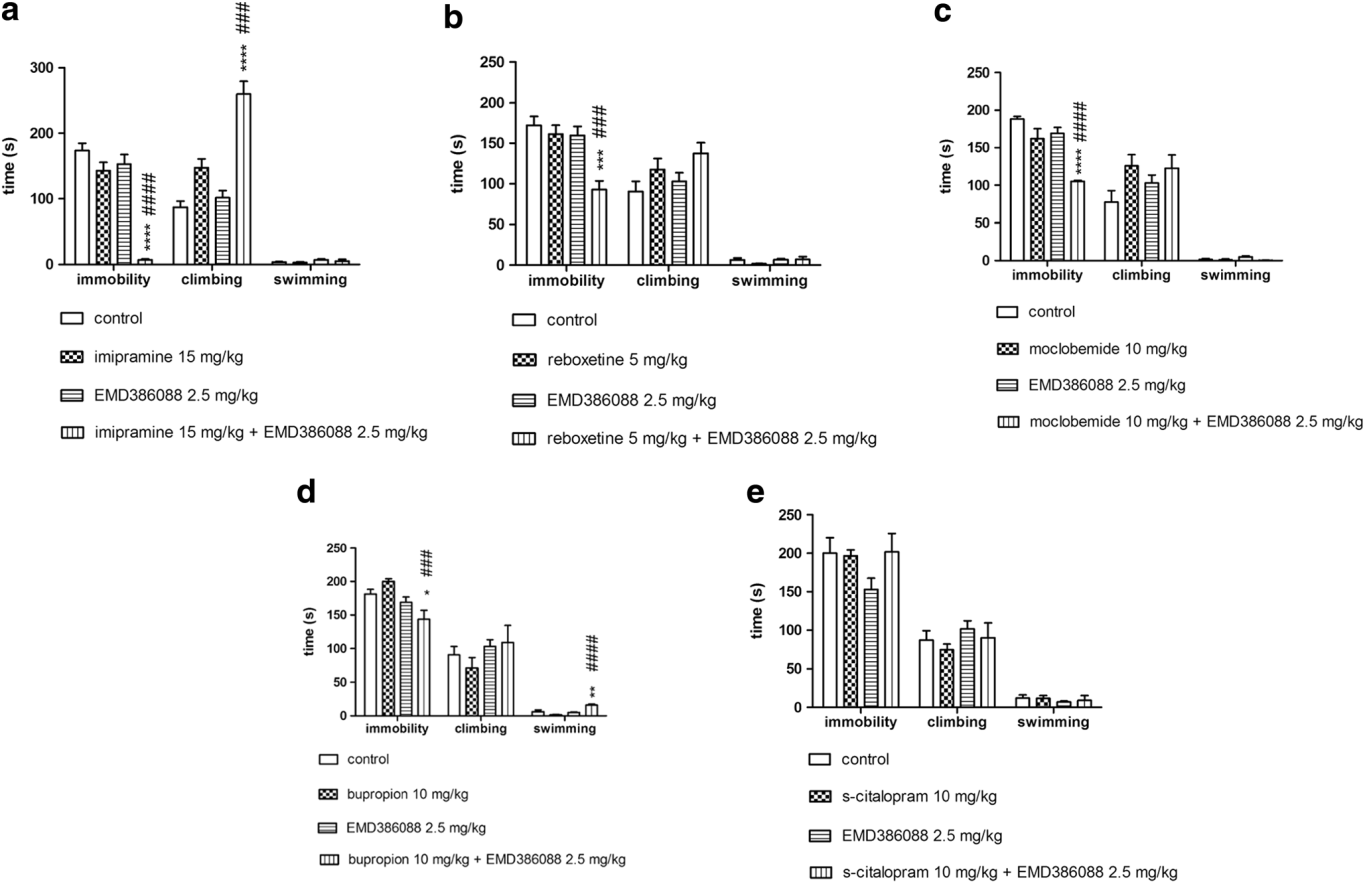
Effects of EMD386088 and antidepressants given alone or in combination in the modified FST in rats. **a** Effects of EMD386088 and imipramine in the FST. **b** Effects of EMD386088 and reboxetine in the FST. **c** Effects of EMD386088 and moclobemide in the FST. **d** Effects of EMD386088 and bupropion in the FST. **e** Effects of EMD386088 and s-citalopram in the FST. EMD386088, imipramine, and s-citalopram were injected i.p. 30 min, while reboxetine, moclobemide, and bupropion were given i.p. 60 min before the test. Values represent the mean ± SEM and were analyzed by two-way ANOVA followed by Bonferroni’s post hoc test: *p < 0,05, **p < 0.01, ***p < 0.001relative to respective vehicle group; ###*p* < 0.001, ####*p* < 0.0001 relative to respective group receiving non-active dose of respective antidepressant drug without EMD386088, *N* = 6–8

The results presented in Fig. 1 show that a concomitant administration of non-active doses of EMD386088 (2.5 mg/kg) and imipramine (15 mg/kg) showed in two-way ANOVA analysis of variance significant decreasing of immobility time (F(1,24) = 23.628, *p* < 0.0001) as well as increasing of climbing behavior (F(1,24) = 12.444, *p* < 0.01) respectively to the vehicle control and single administration of EMD386088 or imipramine (Fig. 1a). EMD386088 (2.5 mg/kg) co-administered with the non-active doses of reboxetine (5 mg/kg, Fig. 1b, (F(1,25) = 6.5843, *p* < 0.05)), moclobemide (10 mg/kg, Fig. 1c, (F(1,25) = 4.8193, p < 0.05)) or bupropion (10 mg/kg, Fig. 1d, (F(1,26) = 6.0071, p < 0.05)) provoked statistically significant interactions only in an anti-immobility effect. A combination of non-active doses of EMD386088 (2.5 mg/kg) and s-citalopram (10 mg/kg) did not produce any significant effect on immobility time, climbing behavior and swimming time in the FST (Fig. 1e, (F(1,24) = 1.9267, NS).

### Effects of EMD386088 and the antidepressant drugs administered alone or jointly in the OF test

The compound EMD386088 (2.5 mg/kg) co-administered with imipramine (15 mg/kg) significantly reduced total distance only evaluated by the OF test, whereas the other parameters, i.e., rearings, X and Y ambulation remained unchanged (Table 1). Other antidepressants given jointly with EMD386088 did not change animals’ locomotor activity in that test. Antidepressants and EMD386088 administered alone had no effect on the exploratory activity in the OF test, except of s-citalopram (10 mg/kg) that significantly reduced total distance (Table 1).

**Table 1:** Effect of EMD386088, imipramine, s-citalopram, reboxetine, moclobemide, and bupropion given alone or in combination on the rat locomotor activity measured in the OF test

Treatment	Dose (mg/kg)	Exploratory activity			
		Total distance (cm)	Rearings	Ambulation X	Ambulation Y
Vehicle + Vehicle	0 + 0	2790 ± 174	72 ± 10	291 ± 40	275 ± 30
Vehicle + imipramine	0 + 15	2212 ± 332	54 ± 3	209 ± 37	203 ± 41
Vehicle + EMD 386088	0 + 2.5	2677 ± 232	90 ± 3	261 ± 29	261 ± 31
imipramine + EMD 386088	15 + 5	1909 ± 125* F(1,20) = 0.4677; *p* = 0.502	89 ± 9 F(1,20) = 1.0969; *p* = 0.307	165 ± 25 F(1,20) = 0.0383; *p* = 0.847	157 ± 30 F(1,20) = 0.2249; *p* = 0.640
Vehicle + Vehicle	0 + 0	2790 ± 174	72 ± 10	291 ± 40	275 ± 30
Vehicle + s-citalopram	0 + 10	1935 ± 121*	64 ± 12	197 ± 15	179 ± 19
Vehicle + EMD 386088 +	0 + 2.5	2677 ± 232	90 ± 3	261 ± 29	261 ± 31
s-citalopram + EMD 386088	10 + 2.5	2160 ± 173 F(1,20) = 0.8898; *p* = 0.357	69 ± 8 F(1,20) = 0.5108; *p* = 0.483	228 ± 26 F(1,20) = 1.1364; *p* = 0.299	232 ± 27 F(1,20) = 1.5337; *p* = 0.230
Vehicle + Vehicle	0 + 0	3102 ± 434	94 ± 13	274 ± 40	281 ± 38
Vehicle + reboxetine	0 + 5	2196 ± 74	84 ± 6	221 ± 16	220 ± 12
Vehicle + EMD 386088 +	0 + 2.5	2721 ± 196	91 ± 4	262 ± 28	261 ± 31
reboxetine + EMD 386088	5 + 2.5	2651 ± 265 F(1,20) = 1.8780; *p* = 0.186	87 ± 6 F(1,20) = 0.0431; *p* = 0.838	267 ± 37 F(1,20) = 0.8117; *p* = 0.378	269 ± 34 F(1,20) = 1.6607; *p* = 0.212
Vehicle + Vehicle	0 + 0	3102 ± 434	94 ± 13	274 ± 40	281 ± 38
Vehicle + moclobemide	0 + 10	2160 ± 183	62 ± 3	200 ± 26	192 ± 23
Vehicle + EMD 386088 +	0 + 2.5	2644 ± 220	89 ± 4	262 ± 35	267 ± 37
moclobemide + EMD 386088	10 + 2.5	2405 ± 168, F(1,19) = 1.5830; *p* = 0.223	69 ± 6 F(1,19) = 0.5090; *p* = 0.484	229 ± 25 F(1,19) = 0.4146; *p* = 0.527	244 ± 30 F(1,19) = 1.0302; *p* = 0.322
Vehicle + Vehicle	0 + 0	2696 ± 308	71 ± 7	251 ± 31	270 ± 40
Vehicle + bupropion	0 + 10	3083 ± 229	83 ± 9	284 ± 31	301 ± 35
Vehicle + EMD 386088 +	0 + 2.5	2722 ± 145	92 ± 9	263 ± 28	262 ± 23
Bupropion + EMD 386088	10 + 2.5	3284 ± 306 F(1,20) = 0.1094; *p* = 0.744	85 ± 6 F(1,20) = 2.0155; *p* = 0.171	365 ± 47 F(1,20) = 0.9526; *p* = 0.341	334 ± 30 F(1,20) = 0.3473; *p* = 0.562

EMD386088, imipramine, and s-citalopram were injected i.p. 30 min, while reboxetine, moclobemide and bupropion were given i.p. 60 min before the test. Values represent the mean ± SEM during 5-min test session compared to the respective group: vehicle + vehicle group *p < 0.05, *NS* not significant (two-way ANOVA is followed by the Bonferroni’s post hoc test), *N* = 6

### Pharmacokinetic study

**Fig. 2:**
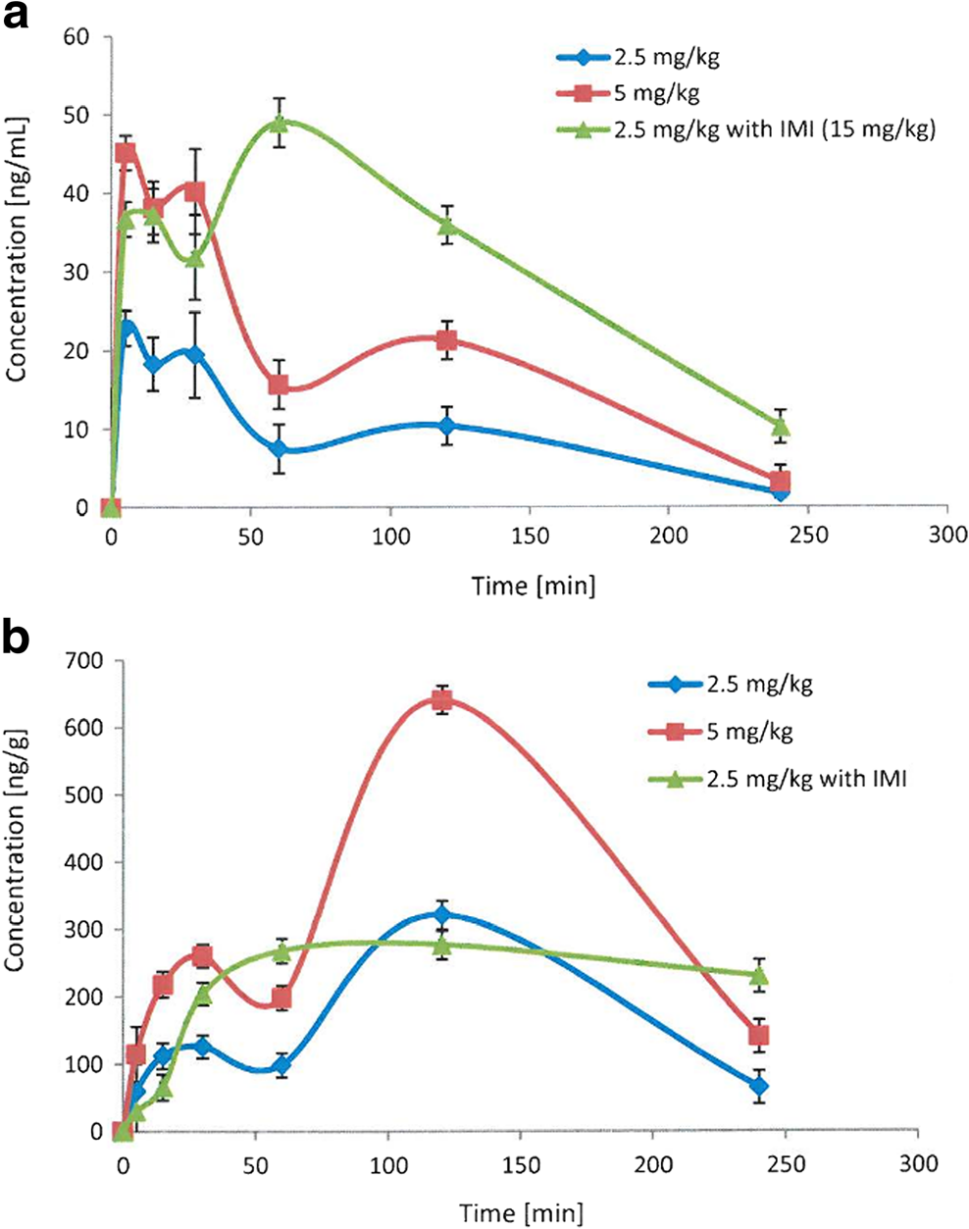
Concentration-time profile of EMD386088 in plasma (**a**) and the brain (**b**) administered alone or jointly with imipramine (IMI) in rats

The plots of mean plasma and brain concentrations versus time profile for EMD386088 administered alone at doses of 2.5 and 5 mg/kg and jointly 2.5 mg/kg with imipramine (15 mg/kg) are depicted in Fig. 2.

The pharmacokinetic parameters for EMD386088 calculated by non-compartmental approach are given in Table 2. The compound was eliminated relatively quickly from rats’ body and its terminal half-life time was about 70 min. EMD386088 showed a large volume of distribution (102 L/kg) that indicates its ability for penetration to the deep compartment. EMD386088 reached the maximum concentration in the blood within 5 min. Coadministration of the compound with imipramine increased the time to reach the maximum concentration (*t*_max_ = 60 min), increased three times the area under the concentration-time curve (AUC), and decreased three times its volume of distribution. The investigated compound exhibited significant distribution to brain which is in agreement with its high volume of distribution but exposition of EMD386088 in the brain after i.p. administration is slow (*t*_max_ = 120 min) (Table 2). The brain to plasma ratio calculated on the basis of AUC_0 → t_ values is very high, ca. 19. Coadministration of EMD386088 with imipramine decreased about two times the brain/plasma ratio (Table 3).

**Table 2:** Pharmacokinetic parameters for EMD386088 administered alone or jointly with imipramine (IMI) in rats

Parameters	Dose [mg/kg]		
	EMD386088 2.5	EMD386088 5	EMD386088 2.5 + IMI 15
AUC_0 → t_ [ng ⋅ min/mL]	2210.6	4527.4	7505.5
t_1/2_[min]	67.3	64.9	77.1
MRT [min]	78.7	78	92.8
*C*_max_ [ng/mL]	22.9	45.2	49
*t*_max_ [min]	5	5	60
*V*_d_/F [mL/kg]	102,183.9	97,140.3	32,204.7
Cl/F [mL/min/kg]	1052.6	1038.8	289.4

EMD386088 and imipramine were injected i.p.

*AUC* area under the curve, *t*_0.5_ terminal half-life, *C*_*max*_ maximum plasma concentration, *t*_*max*_ time to *C*_max_, *V*_*d*_ */F* apparent volume of distribution, *Cl/F* apparent systemic clearance, *MRT* mean residence time

**Table 3:** Distribution of EMD386088 administered alone or jointly with imipramine (IMI) in rats’ brain

Parameters	Dose [mg/kg]		
	EMD386088 2.5	EMD386088 5	EMD38688 2.5 + IMI 15
AUC_0 → t_ [ng ⋅ min/g]	41,936.2	84,462.8	56,428.6
MRT [min]	114	114.8	127.2
*C*_max_ [ng/g]	321	640.3	276.9
*t*_max_ [min]	120	120	120

EMD386088 and imipramine were injected i.p.

*AUC* area under the curve; *MRT* mean residence time; *C*_*max*_ maximum plasma concentration; *t*_*max*_ time to *C*_max_

### Metabolic stability of EMD386088 study

**Fig. 3:**
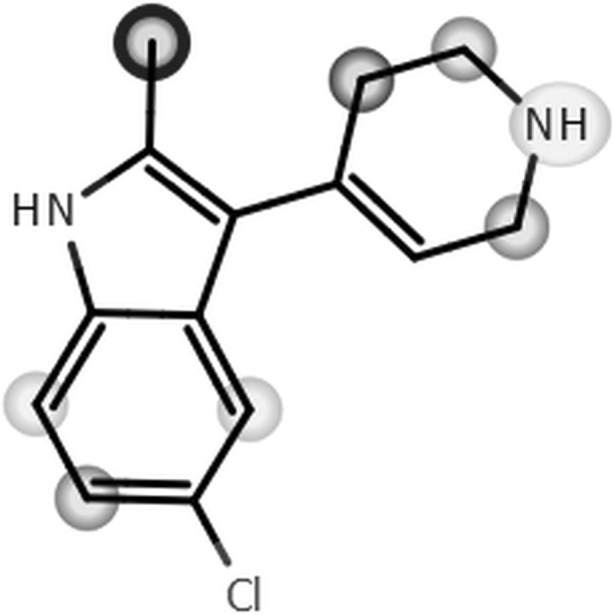
The plot of MetaSite predictions for sites of metabolism of EMD386088

The metabolic stability of EMD386088 was examined first in silico by using MetaSite software (Cruciani et al. 2005). The plot of MetSite predictions for the most probable sites of metabolism of EMD386088 by liver computational model is shown in Fig. 3.

The darker gray color of the marked functional group indicates its higher probability to be involved in the metabolism pathway. The black circle marked the site of EMD386088 involved in metabolism with the highest probability.

**Fig. 4:**
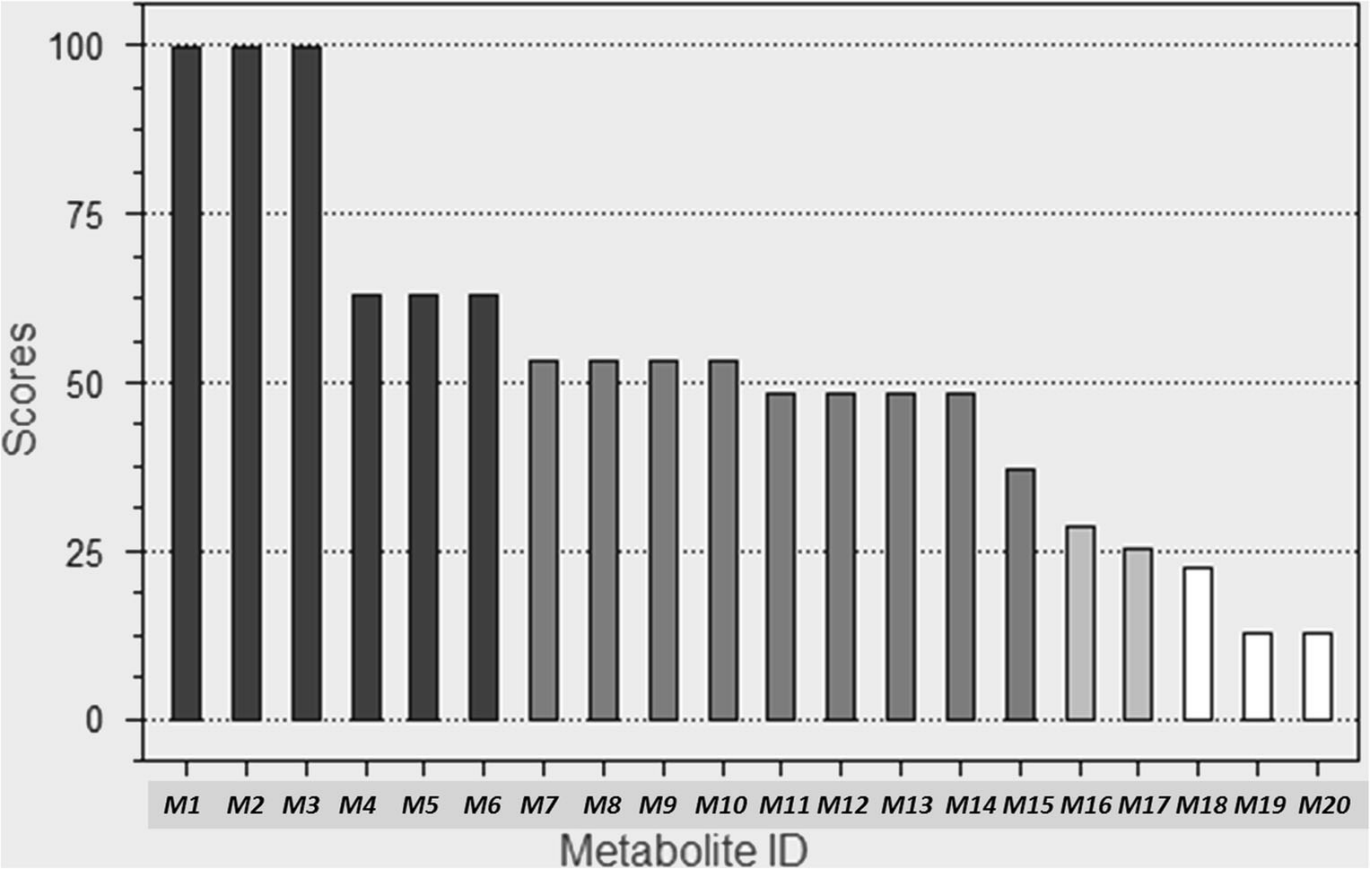
EMD386088 metabolites ranking generated by MetaSite software

**Fig. 5:**
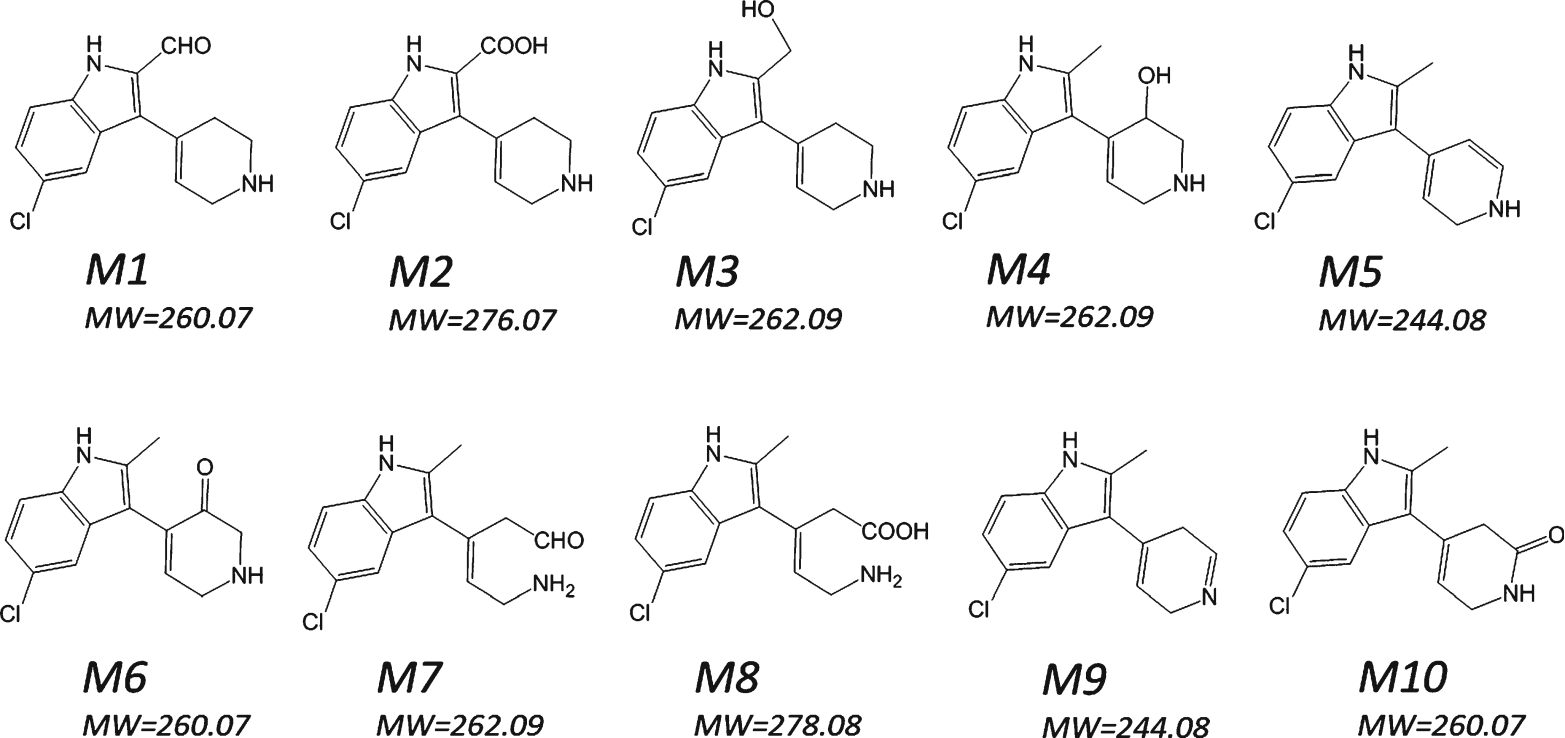
Structures of 10 most probably (scores > 50%) metabolites of EMD386088 generated by MetaSite software

MetaSite is also able to predict the most probably structures of metabolites. The metabolites ranking and the structures of 10 metabolites which may occur with the highest probability (> 50%) are presented on Figs. 4 and 5.

**Fig. 6:**
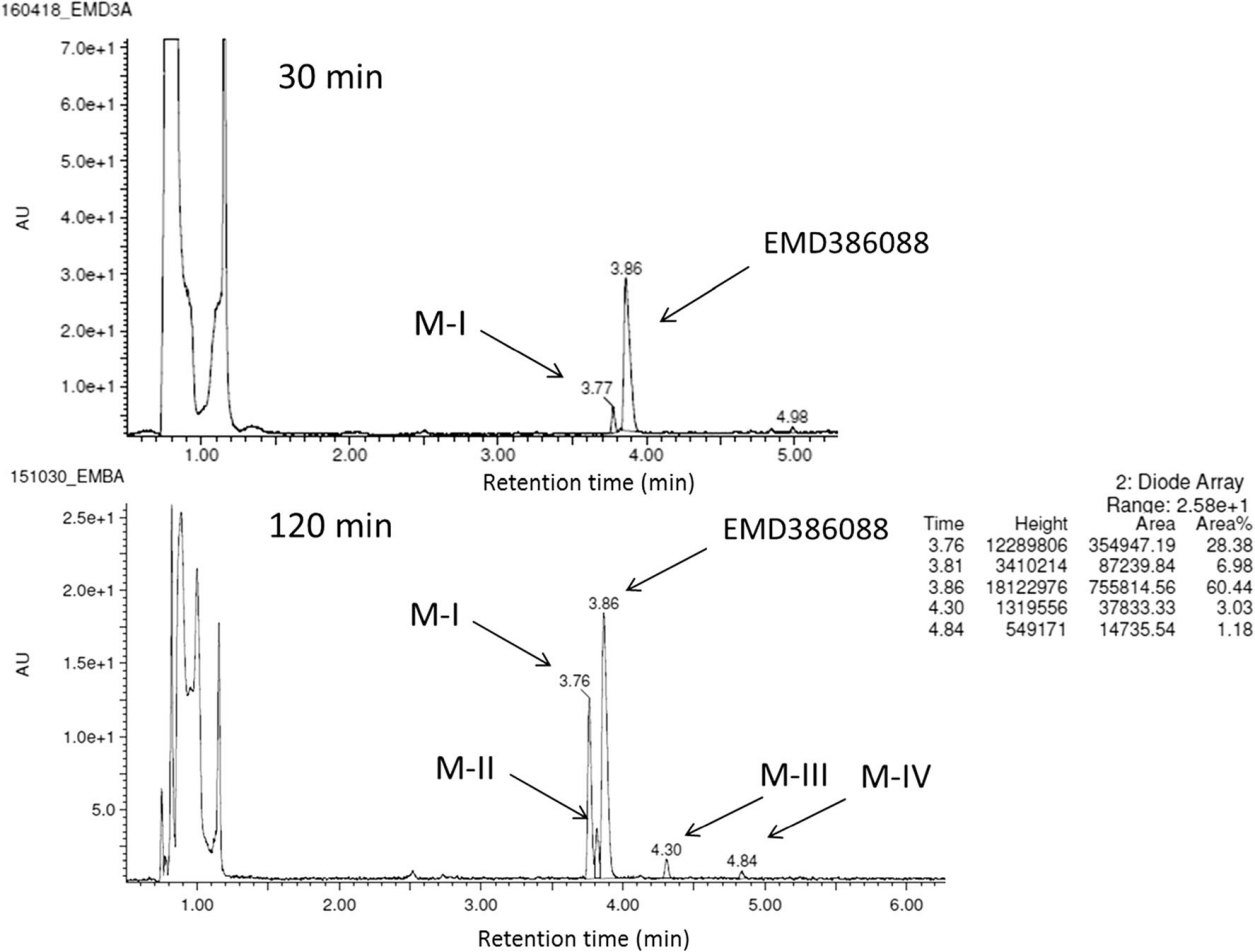
The UPLC spectra after 30 and 120 min reaction of EMD386088 with RLMs. *UPLC* ultra-performance liquid chromatography, *RLMs* rat liver microsomes

**Fig. 7:**
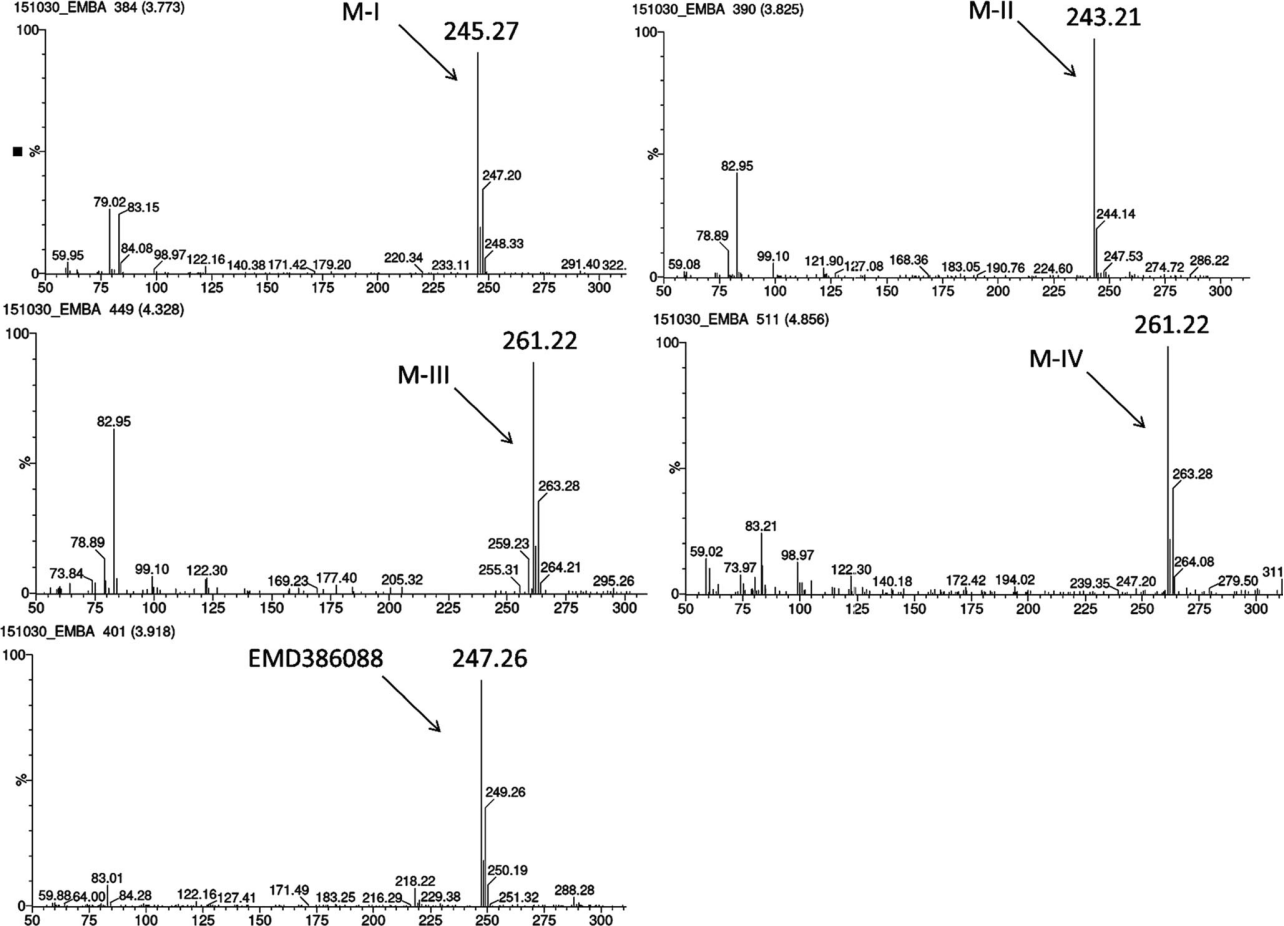
MS spectra of EMD386088 and its metabolites in the total ion chromatogram after 120 min of incubation with RLMs. *MS* mass spectrometer, *RLMs* rat liver microsomes

The metabolic stability of EMD386088 was also evaluated in vitro using RLMs. A full scan chromatogram of the reaction mixture after 30 min of EMD386088 incubation showed the presence of one metabolite M-I, whereas after 120 min its four metabolites (M I-IV) occurred (Figs. 6 and 7). Moreover, as was shown at the UPLC spectrum (Fig. 6), EMD386088 seems to be rather a metabolically unstable molecule, because almost 40% of the compound was metabolized in the presence of RLMs after 120 min.

The LC/MS analysis provided the accurate molecular masses of all EMD386088 metabolites which were up from m/z = 247.26 to m/z = 261.22 corresponding to the quasimolecular ions [M + H]^+^ of metabolites M-III and M-IV or down to m/z = 245.27 (M-I) or m/z = 243.21 (M-II) (Fig. 7).

**Fig. 8:**
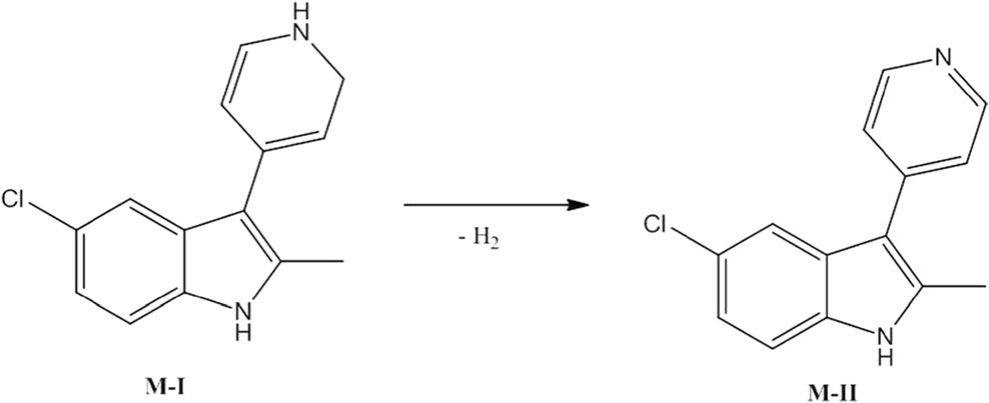
Proposed structure of metabolite M-II of EMD386088

The most probable structural formulas of metabolites proposed by MetaSite program were compared with the masses of pseudomolecular ions from LC/MS analysis. Regarding obtained by RLMs, the main EMD386088 metabolite M-I, its molecular mass corresponds to the proposed structure of *M5* (Fig. 4) obtained by the dehydrogenation of tetrahydropyridine moiety. The molecular mass of metabolite M-II was not found in in silico data, however, this metabolite seems to be the effect of succeeding dehydrogenation of metabolite M-I (Fig. 8).

The molecular masses of metabolites M-III and M-IV correspond to the predicted in silico structures of *M1*, *M6*, and *M10* (Fig. 4) which are the effect of EMD386088 oxygenation either in the methyl substituent or in the tetrahydropyridine moiety.

### Effect of EMD386088 on CYP3A4 and CYP2D6 activity

**Fig. 9:**
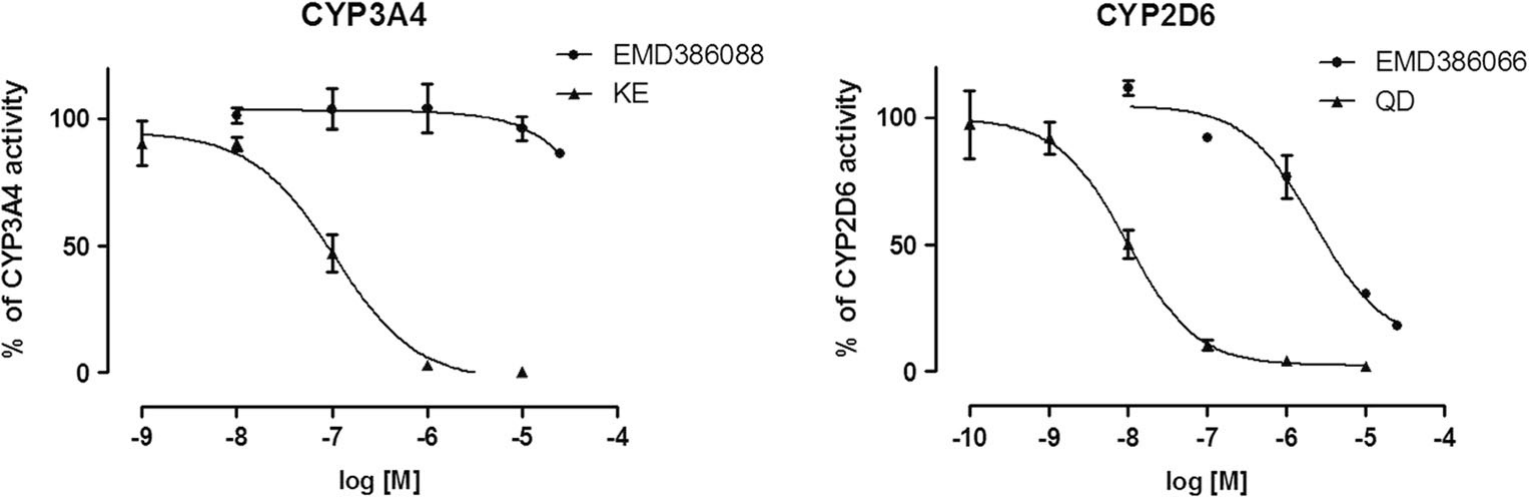
Effects of EMD386088 on recombinant human CYP3A4 and 2D6 P450 cytochromes activity

The additional study was performed to determine the effect of EMD386088 on CYP3A4 and 2D6 activity. The strong CYP3A4 inhibitor ketoconazole (KE *IC*_*50*_ = 0.1 μM), and strong CYP2D6 inhibitor quinidine (QD *IC*_*50*_ = 0.01 μM) were used as references. EMD386088 did not influence CYP3A4 activity, and was determined as a very weak CYP2D6 inhibitor (EMD386088 *IC*_*50*_ = 2.25 μM) (Fig. 9). Thus, the effect of EMD386088 on CYPs 3A4/2D6 may be rather excluded as a potential reason responsible for the observed activity of EMD386088 co-administered with antidepressants.

## Discussion

The major finding of the present study was that the inactive dose of a partial agonist of 5-HT_6_ receptor EMD386088 co-administered with an inactive dose of some antidepressants (i.e., imipramine, moclobemide, reboxetine, and bupropion) induced pronounced anti-immobility effects in rats. Only during coadministration of inactive doses of EMD386088 and s-citalopram antidepressant-like activity in FST has not been observed. Moreover, all the above-described positive interactions seem to be specific, since there was no increase in exploratory activity of rats after administration of the investigated compounds (single or combined).

The FST is the most extensively used behavioral procedure for detecting the potential antidepressant activity of compounds. The modified version of FST has been shown to reliably detect the antidepressant activity of selective serotonin reuptake inhibitors (SSRIs) as well as other compounds that produce their effects by activating the 5-HT system (Detke et al. 1995; Cryan et al. 2005). The modified FST showed that swimming behavior was increased by SSRIs, while climbing behavior was increased by antidepressants with a selective impact on catecholamine transmission (Detke et al. 1995; Cryan et al. 2005). The most potent antidepressant-like activity in FST was observed after coadministration of non-active doses of EMD386088 and imipramine (a significant increase in the climbing behavior and a decrease in immobility, compared to single administration as well as to control group). Imipramine acts as a non-selective 5-HT and noradrenaline reuptake inhibitor (Schloss and Williams 1998; Slattery et al. 2004). Our previous study has revealed that EMD386088 did not show any significant effect on 5-HT and its intraneuronal metabolite (5-hydroxyindoleacetic acid) concentrations in brain structures (striatum, nucleus accumbens, and hippocampus) of different rats. Moreover, Ward et al. (1995) found a 5-HT_6_ receptor mRNA in 5-HT projection fields rather than in region containing cell bodies, which suggests post-synaptical localization of 5-HT_6_ receptors, and Gérard et al. (1996) reported that 5-HT_6_ receptors are located outside 5-HT neurons. Our results are in line with above-presented data. Activation of 5-HT_6_ receptors by EMD386088 does not promote the antidepressant-like activity of s-citalopram in rats.

Besides the serotonergic and noradrenergic neurotransmission, the dopaminergic pathways may also be involved in the pathomechanism of depression (Randrup and Braestrup 1977). This system is associated with serotonergic, GABAergic, cholinergic, and glutamatergic neurotransmission and interactions between them contribute to changes in their mutual signaling. Our previous neurochemical data from ex vivo experiments as well as behavioral data showed that anti-immobility activity of EMD386088 may be connected with the activation of dopaminergic system, while neither noradrenergic nor serotonergic systems are involved in its effect. EMD386088 also possesses a significant affinity for dopamine transporter, which may be the mechanism responsible for the abovementioned effect (Jastrzębska-Więsek et al. 2016). Thus, the most effective activity observed for coadministration of the 5-HT_6_ receptor partial agonist and imipramine depends on the involvement of three neurotransmitter systems, i.e., dopaminergic from EMD386088 side and serotonergic and noradrenergic from imipramine side unless pharmacokinetic interaction plays an important role here.

Pharmacokinetic drug–drug interaction occurs when one drug changes the concentration of another by affecting its absorption, distribution, metabolism, or excretion (Corrie and Hardman 2014). To the best of our knowledge, there are no literature data about pharmacokinetic properties of EMD386088. Therefore, to evaluate the physical properties of EMD386088 and its interaction with imipramine, some pharmacokinetic parameters as well as brain and serum concentrations of both compounds, administered i.p. to rats, were determined. The pharmacokinetic study in vivo has revealed a rapid absorption of EMD386088 and a large volume of distribution, which indicates its penetration into the peripheral compartments after i.p. administration. Furthermore, EMD386088 administered individually penetrates the blood–brain barrier with a high brain/plasma ratio. These data are in line with the present and our previous findings. A dose of 2.5 mg/kg is not active in the FST and produced a lower brain concentration in the brain of rat. Our earlier data indicated that EMD386088 at a dose of 5 mg/kg i.p. exerts an antidepressant-like effect in the FST in rats (Jastrzębska-Więsek et al. 2015). This has been confirmed by at least a double increase in the concentration of EMD386088 in brain. Coadministration of EMD386088 and imipramine resulted in a slower absorption of the compound, decrease in the volume of distribution, and decrease in penetration by 2.5 fold.

Most of the isoenzymes of cytochrome P450 are involved in the metabolism of antidepressant drugs, especially CYP3A4 and CYP2D6 (Spina et al. 2008). Hence, if changes in the brain concentration of an antidepressant are observed after coadministration with another drug, there might be an inhibition of enzymes involved in the biotransformation. But, the in silico and in vitro studies on the metabolic stability of EMD386088 showed the dehydrogenation of tetrahydropyridine moiety as its main metabolic pathway. Furthermore, EMD386088 did not influence on CYP3A4 activity, and has been classified as a very weak CYP2D6 inhibitor. Moreover, no increase in brain concentration of imipramine+EMD386088 was noted. The increase in antidepressant-like activity of these drugs was most likely related to another possibility. EMD386088 was characterized with a very high permeability for the brain. It is well documented that amphiphilic or basic (pKa > 8) lipophilic drugs are extensively metabolized and can be taken up by acidic compartments like lysosomes. Lysosomes constitute the largest and the most important acidic cell compartment (pH = 4–5) and play a significant role in pharmacokinetics. Weak bases in non-ionized state diffuse through biological membranes and accumulate in the acidic interior of lysosomes, where they are protonated and become unable to diffuse back into cytosol, which leads to the accumulation of a drug (Walczak 2013). Lysosomes are present in lungs, liver, kidneys, spleen, leukocytes, and macrophages and in smaller quantities in the brain, heart, muscles, and adipose tissue. The process of lysosomal trapping is saturable, energy-dependent, and requires cellular integrity. In the present study, coadministration of EMD386088 with imipramine decreases the compound permeability to brain. Imipramine is known as an inhibitor of lysosomal trapping (Daniel and Wojcikowski 1999). A substantial decrease of EMD386088 concentration in lysosomes (depot form) observed in vivo leads to an increase in its plasma concentration which, in turn, decreases the compound’s volume of distribution.

## Conclusions

In summary, the activation of 5-HT_6_ receptor may facilitate antidepressant-like activity of some antidepressants, whose mechanism of action is connected with dopaminergic and noradrenergic transmission. The effect of EMD386088 on CYP3A4 and CYP2D6 may be rather excluded as a potential reason responsible for the observed interactions. The distribution kinetics of EMD386088 is driven by the compound lipophilicity and uptake into lysosomes, and these phenomena provide a possible basis for the compound interaction with other drugs, e.g., antidepressants in preclinical studies.
